# Cancer stem cells and oral cancer: insights into molecular mechanisms and therapeutic approaches

**DOI:** 10.1186/s12935-020-01192-0

**Published:** 2020-04-07

**Authors:** Ghazaleh Baniebrahimi, Fatemeh Mir, Razieh Khanmohammadi

**Affiliations:** 1 Department of Pediatric Dentistry, School of Dentistry, Tehran University of Medical Sciences, Tehran, Iran; 2 Department of Pediatric Dentistry, School of Dentistry, Zahedan University of Medical Sciences, Zahedan, Iran

**Keywords:** Oral cancer stem cells, Pathogenesis, Molecular pathways

## Abstract

Cancer stem cells (CSCs) have been identified as a little population of cancer cells, which have features as the same as the cells normal stem cells. There is enough knowledge of the CSCs responsibility for metastasis, medicine resistance, and cancer outbreak. Therefore, CSCs control possibly provides an efficient treatment intervention inhibiting tumor growth and invasion. In spite of the significance of targeting CSCs in treating cancer, few study comprehensively explored the nature of oral CSCs. It has been showed that oral CSCs are able to contribute to oral cancer progression though activation/inhibition a sequences of cellular and molecular pathways (microRNA network, histone modifications and calcium regulation). Hence, more understanding about the properties of oral cancers and their behaviors will help us to develop new therapeutic platforms. Head and neck CSCs remain a viable and intriguing option for targeted therapy. Multiple investigations suggested the major contribution of the CSCs to the metastasis, tumorigenesis, and resistance to the new therapeutic regimes. Therefore, experts in the field are examining the encouraging targeted therapeutic choices. In spite of the advancements, there are not enough information in this area and thus a magic bullet for targeting and eliminating the CSCs deviated us. Hence, additional investigations on the combined therapies against the head and neck CSCs could offer considerable achievements. The present research is a review of the recent information on oral CSCs, and focused on current advancements in new signaling pathways contributed to their stemness regulation. Moreover, we highlighted various therapeutic approaches against oral CSCs.

## Introduction

It is widely accepted that the head and neck cancers involve above 650,000 people and 330,000 mortality each year throughout the world [1]. For example, the head and neck cancers involve 3% of the malignancy with ~ 53,000 Americans, whose head and neck cancers developed every year and 10,800 who died due to this diseases in the United States [2]. Moreover, about 250,000 people (estimation showed 4% of the cancer occurrence) and 63,500 deaths have been reported in Europe in 2012 [3]. In addition, the commonest malignancy has been considered to be the squamous cell carcinoma (SCC) and the respective variants.

According to a study in the field, diets, oral hygiene, carcinogen exposure, family history, infectious agents, as well as the pre-existing medical conditions contributed separately or jointly to the HNSCC progression. Among the mentioned cases, tobacco smoking has been completely shown as one of the predominant risk factors for HNSCC and such as risk had a correlation to duration and intensity of smoking. It has been also found that quitting the smoking reduced but did not overcome the risks of the cancer expansion [4]. In addition, environmental exposure to the smoking of tobacco; that is, passive smoking apparently enhanced the risks of the HNSCC progression even for people who had at all experienced active smoking [4].

Studies also showed the use of heavy alcohol as one of the independent risk factors for HN-SCC, especially for hypopharynx cancer [5]. Even though exposure to tobacco and use of alcohol involved in most HNSCCs occurring in larynx, hypopharynx, and oral cavity, their contribution to the oropharynx tumorigenesis was minor. On the other hand, authors approved the oncogenic HPV, specifically Type 16, as one of the causes in about 70% of the oropharyngeal cancers [6]. However, as the cigarette smoking declined in several regions worldwide, the HPV-16 infection would be the greatest risk factor, shifted of HNSCC demographic towards younger patients with no experience of smoking or drinking. Actually, conventional risk factors like exposure to alcohol and tobacco, had no contribution to the HPV-mediated carcinogenesis of oropharynx [6]. Put differently, HPV-related HNSCC showed a close correlation to the oral HPV infection and specific sexual positions, which facilitated iterative viral exposure, including the early age of the sexual debuts, the increased numbers of the life-time oral sexual and vaginal partners, recurrent oral-anal and oral-genital contacts, as well as rare application of the barrier in the course of sexual activities [7]. Even though researchers have enough data of the risk factors for the viral transmission, they emphasize the ones related to the consequent HPV-induced tumorigenesis. Moreover, authors suggested specific situations and behaviors, which altered the anti-tumor immunity, as the potent parameters affecting the viral persistence and tumor expansion. Long examined carefully as one of the potent sources of the DNA-damaging carcinogens. It has been shown that smoking marijuana could have higher relationship with progressing the HPV-positive HNSCCs for its immuno-regulatroy impacts. Therefore, cannabinoids bound to CB2 receptor expressed on the B-cells, T-cells, NK-cells, dendritic cells, and macrophages in the humans’ tonsillar tissues. Hence, binding had been capable of suppressing the immune response, diminishing the host response to the viral pathogen, and attenuating antitumor activities [8–10]. Consequently, using the marijuana might influence each stage of the HPV-induced tumorigenesis from the time of infection initiation to the viral persistence, tumor induction, metastasis, and growth [11].

In terms of clinical examinations, organized lesions, including leukoplakia, which are histologically categorized as the non-dysplastic or dysplastic leukoplakia, frequently come before head and neck cancer. Nonetheless, researchers characterized dysplastic leukoplakia as one of the oral pre-malignant lesions related to a possibly development to cancers. Nonetheless, the researchers did not regard dysplastic leukoplakia as one of the precise predictors of the cancer risks [12, 13]. Several therapeutic approaches i.e., cell therapy, gene therapy, nanotechnology-based therapies, utilization of natural compounds are used in the treatment of different cancers such as oral cancers [14–22]. In general, it is possible to manage initial-phase tumors via radiotherapy and surgery; however, successful therapy has an inverse proportionate to the extent of disease while treating. If chemotherapy and radiation treatment are combined, even though they affect the treatment of 97% of the initial-phase tumors, they just 33% affect the treatment of the advanced tumors [23].

Current research discovered and verified the patho-physiologic contribution of CSCs that are also defined as tumor-initiating cells in the lengthy maintenance of cancers [24, 25]. It is widely accepted that CSCs are little sub-populations of tumor cells sharing several molecular similarities to the embryonic and normal adult stem cells. Some researchers separated CSCs from a variety of main tumors and created cancer cell lines such as OSCC [26–32]. These CSCs contribute crucially to the metastasis, tumorigenicity, and recurrence. For this reason, they are regarded as the origin of the cancer. Hence, it is necessary to increase knowledge of the molecular features and signaling paths specific to the oral CSCs in order to develop new targeted and efficient treatments for head and neck cancer.

## Oral cancer stem cells isolation

The first reports of the presence of a CSC population has been related to the leukemic cells [33]. Positive and negative staining of the leukemic CSC population has been done with CD34 and CD38 (CD34^+^/CD38), respectively. They were able to develop leukemia while inoculating onto immunocom-promised mice. Afterwards, researchers have widely investigated the CSC populations, and substantially determined them in different solid tumors like the prostate, neck, pancreas, colon, head, brain, and breast [34–37]. The existence of sub-populations of oral CSCs has been primarily proposed by the study, which showed just a sub-population of OSCC cells is able to create a developing tumor mass [38]. Chiou et al. showed that a sub-population of OSCC cells extracted from the cultivated OSCC cell lines have features of the two stem cells and invasive malignant tumors such as self-renovation, tumorigenic potentials, migratory abilities, and radio-resistance [26]. Multiple researchers published information of the successful separation of oral CSC populations via different markers [26–32, 39, 40]. Generally, cancer stem cells in OSCC are possibly separated via the cell-surface markers or the respective specific practical features [41–43]. Yet, any specific marker and CSC feature cannot particularly isolate oral CSCs populations from OSCC cells, which demonstrate that CSC populations are heterogeneous [44, 45]. Hence, it would be crucial to identify further oral CSC markers and the respective cellular features.

Several research found differential expression of CD44 on cancer stem cells against non-cancer stem cells in different solid tumors [46, 47]. These CD44^+^ CSC populations have been substantially isolated from the head and neck cancers via flow cytometry sorting through CD44 antibody [34, 39, 40]. CD44 that is a multi-functional trans-membrane glycoprotein attaches to hyaluronan. It acts as an essential surface molecule, which is capable of interacting with different intrinsic and extrinsic signals for regulating several gene expressions. It is widely accepted that CD44^+^ cells may be fractionated from heterogeneous single-cell-prepared cancer cells via CD44 specific antibody labeling accompanied by the flow cytometry arrangement [48]. Such CD44^+^ cells have definite features of the stem cells such as self-renovation capacities, great tumorigenic potentials, metastasis, and resistance to medicine [46, 47]. If the CD44 is inhibited under experimental conditions, CD44^+^ CSCs decline their stemness features, which shows expressing CD44 is crucial to preserve the CSC phenotype [46].

Researchers applied ALDH1 activity as a cancer stem cell marker for a variety of cancers such as OSCC [49–51]. It is a cytosolic iso-enzyme that has control over the oxidation of intra-cellular aldehydes. It involves in the retinol oxidation to retinoic acid in the initial stem cell differentiation [52]. One of the subpopulations of the cancer cells with improved CSC activities indicates great activities of ALDH1 (ALDH1^HIGH^ or ALDH1^+^) in comparison with the non-CSC population, indicating that ALDH1^+^ cells are possibly an origin of CSCs [27, 53, 54]. In addition, ALDH1^HIGH^ cancer cells have higher CSC traits than the ALDH1^low^ cells [34, 51, 55]. Targeting ALDH1 largely inhibits several CSC features in human cancer cells [56]. Nonetheless, there is still controversy of the issue if CD44 or ALDH1 itself may be described as one of the unique molecules for identifying cancer stem cells or not. Therefore, researchers usually employed combining ALDH1 and CD44 as one of the markers to separate cancer stem cells in the head and neck cancer cells [34]. In fact, ALDH1^HIGH^/CD44^+^ cancer cells have higher CSC features than the ALDH1^low^/CD44^−^ cancer cells [57, 58], illustrating that combining CSC markers can ameliorate CSC isolation specificity.

CSCs are able to improve themselves in the nonadherent tumor spheres cultivated in ultra-low binding plates for supporting un-differentiated development of self-renovating stem cells [59]. Researchers found that the sphere medium would be in a serum-free situation complemented with sufficient mitogens, including fundamental fibroblast development agent and epidermal growth factor (EGF) [60–62]. Plentifulness and growth kinetics of the tumor spheres represent self-renovation capacities in a certain cultivation of heterogeneous cancer cells, which indicates the contents of cancer stem cells. Therefore, researchers proposed that the tumor sphere-forming assay would be one of the popular techniques employed for isolating cancer stem cells from heterogeneous cancer cell populations via certain practical property of cancer stem cells. It should be noted that the tumor sphere-forming cells detected in several major tumors and cultured cancer cell lines showed higher features of cancer stem cells in comparison with the features of the relative adherent mono-layer cells, which have been regarded as non-CSCs [63]. It has been known that the tumor sphere-forming cells enjoy the increased tumorigenicity, metastatic potentials, and medicine resistance as well as influential expression of stemness agents, which indicates their crucial contribution to pathogenezing and progressing cancers [64–69]. As such, tumor spheres extracted from OSCC cells show higher stem-like features. They show greater volume of expression of pluripotent transcription agents such as Lin28, Nanog, KLF4, Oct4, and Sox2 in comparison to the respective adherent mono-layer cells [26, 60, 70, 71]. Moreover, influential expression of CSC specific markers, including CD44 and ALDH1 is expressed by oral tumor sphere-forming cells [60, 61, 72]. These would be largely tumorigenic as inoculated into nude mice and retain the respective self-renovation capacities for several generations [72]. Table 1 listed oral cancer stem cell markers.

**Table 1:** Oral cancer stem cell markers

Type of head and neck cancer	Marker	Refs
HNSCC cell lines	ALDH	[73]
HNSCC cell lines	ZsGreen-cODC	[74]
HNSCC cell lines	CD10	[75]
Nasopharyngeal SCC cell lines	Side population	[76]
Laryngeal SCC cell lines	CD133^+^	[77]
Glottic carcinoma biopsy	CD29	[78]
Laryngeal SCC cell lines	CD44^+^	[79]

## Oral cancer stem cells and their signaling pathways

**Fig. 1:**
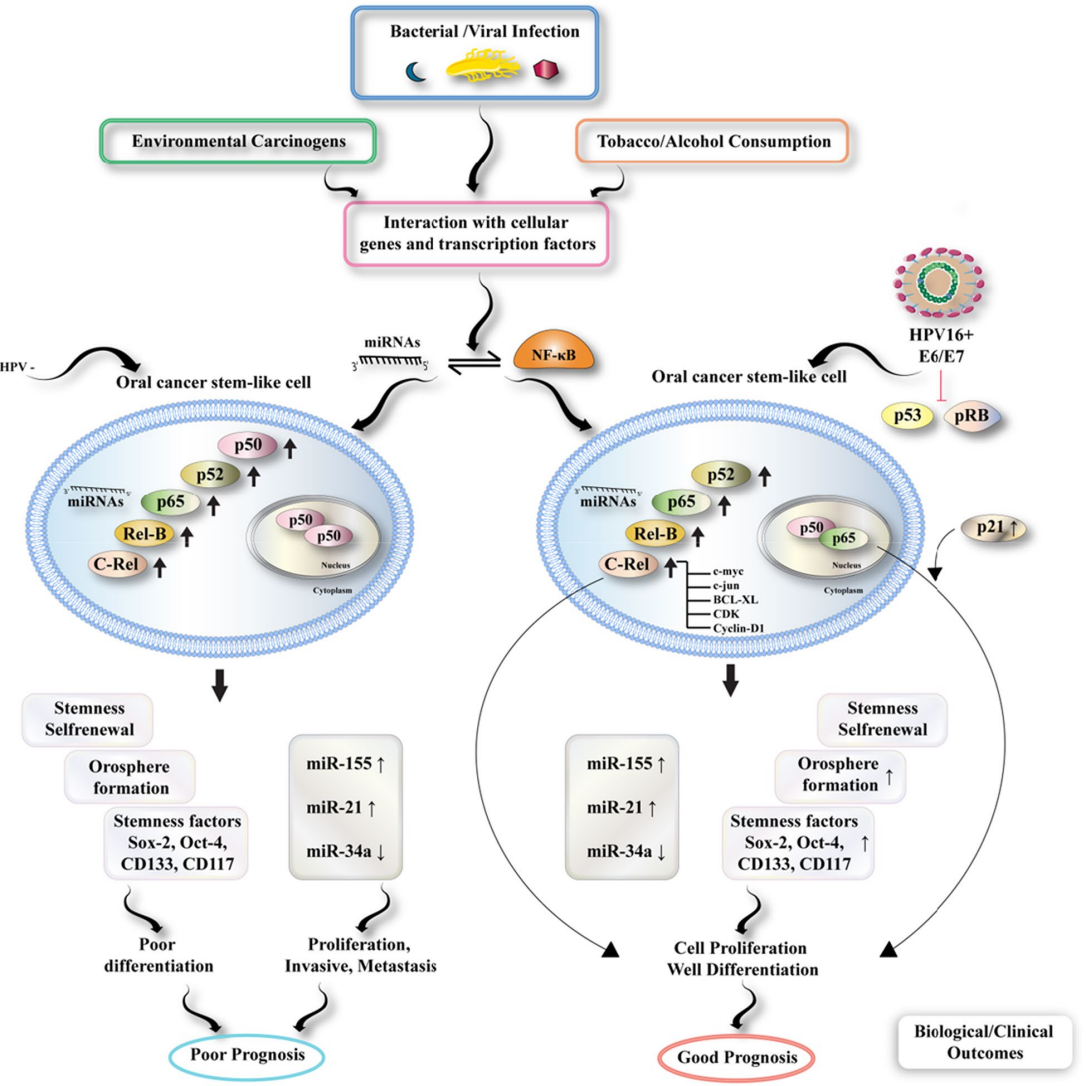
The scheme representing the contribution of NF-κB and miRNA to OCSCs and regulation in the presence or absence of HPV16 infection and their interactions with additional gene products resulting in acceptable or the worst prognosis when given treatment

CSCs have the shared features with normal stem cells and several certain traits maintaining tumor growth and invasion. One of the primary features of CSCs is their self-renewal capacities, so that it apparently is one of the motives to begin and maintain tumorigenicity [24]. CSCs Self-renewal may be retained through multiple endogenous signaling paths, including Wnt, Bmp, Pten, Notch, B cell–specific Moloney murine leukemia virus integration site 1 (Bmi1), TGF-β, and Hedgehog [80–86] that would be often actuated in human cancers [85, 87, 88]. A variety of signalling pathways and molecules could be involved in oral CSCs (Fig. 1). In the below, we have summarized some of them.

### EMT

The CSCs unique feature is their metastatic potentials [24]. It has been found that EMT confers migratory potentials in cancer cells, and this procedure involves essentially in cancer metastasis. EMT is a procedure, through which epithelial cells would lose the respective properties for gaining mesenchymal phenotype, and therefore result in migrating and invading cells [89, 90]. During EMT, expressing the epithelium-specific protein; for example, cytokeratins and E-cadherin would decline, while expressing mesenchymal-specific proteins such as vimentin, fibronectin, and N-Cad increase. Researchers determined the major transcription parameters for EMT such as TWIST, LEF-1, SNAIL, and overexpressing these factors enhanced EMT [91, 92]. Fractionated CSCs would over-express EMT transcription factors, and considerably illustrate in vivo metastatic potentials in comparison with the ones in un-fractionated cancer cells, which suggests that cancer stem cells would be a key origin of the metastatic cancer cell population [93]. Additionally, some studies indicated vital contributions of the zinc-finger E-box–binding homeobox (Zeb) to maintain the features of the EMT and cancer stem cells [94]. Zeb1 and Zeb2 remarkably enhanced cancer stem cells in the head and neck in comparison with the ones in non-CSCs [95]. Zeb1 and Zeb2 knock-down in the head and neck cancer cells diminished CSC features, including emigration, self-renovation capacities, and stemness markers expressions. Furthermore, their inhibition suppressed growing in vivo tumor and level of metastasis to remote locations [95]. Contrarily, co-overexpressing Zeb1 and Zeb2 elevated the emigration capability of the head and neck cancer cells [95].

### ABC transporters

It is possible to enrich CSC population following chemo-radiotherapy, which suggests that treatment leads to chemo-radioresistance, and or selectively improves the resistant cell population. Moreover, researchers documented different molecular determining factors for CSC chemo-radioresistance. However, they completely agreed that contribution of adenosine triphosphate (ATP)-binding cassette (ABC) transporters would be the major actors in resisting treatment [96]. ABC transporters are the membrane transporters, which are capable of pumping different little molecules (e.g., anti-cancer medicines) out of the cells at the expense of ATP hydrolysis, and thus led to the decreased intra-cellular medicine concentrations. Overexpressing ABC transporters is one of the popular occurrences found in multi-drug resistance in cancers [97]. The increased levels of ABC transporters are expressed by normal cells. Overexpressing ABC transporters in cancer cells enhanced their chemo-radioresistance [98]. Suppressing ABC transporters elevates anti-cancer medicine sensitiveness in cancer [99]. The research usually indicated that ABC transporters are actually major molecular determining factors of CSC chemo-radioresistance. Little populations of CSCs that have higher efflux capacities because of the higher ABC transporters are possibly isolated by treating the cells with Hoechst 33342 dye. Afterwards, they would be determined as a side population (SP). Several research revealed substantial separation of CSCs through the above method, and SP cells have higher capacitues for the CSC phenotype compared to the non-SP cells [100, 101]. Researchers also found the existence of SP cells in oral SP cells and oral cancer cells in comparison to the non-SP cells. They enhanced anti-cancer medicine resistance and the stem cell phenotype [100, 102, 103]. Hence, it is generally agreed that CSCs originally resist to chemo-radiotherapy and involve in tumor relapse [25].

### Inflammatory molecules

As the abnormal actuation and over-expression of the pro-inflammatory transcription agent, NF-κB contributes importantly to the regulation of different cellular procedures such as apoptosis, cell differentiation, signal transduction paths, and transformation, particularly over the development and metastasis process of multiple cancers such as oral cancer, unpaving the contribution of NF-κB proteins is of high importance [104]. Studies revealed that the NF-κB path would be actuated commonly in the cancer and cancer stem cells of various malignances such as leukemia, ovary, breast, glioblastoma, pancreatic, prostate, and colon cancers. Notably, its actuation induces radiotherapy and chemotherapy resistance [105–107]. Additionally, miRNAs are still the other significant modulatory molecule engaged during carcinogenesis. They also may function as oncogenes or tumor inhibitor genes so that they practically interplay with NF-κB and additional molecules. However, there is not enough knowledge of the NF-kB and miRNA in strong relationship with crucial risks, alcohol, tobacco, and the increased risks of HPV infections during oral carcinogenesis and its prognoses. Bano et al. separated cancer stem-like SP cells from HPV ± ve OSCC cell lines, and the main tumors, forming orospheres, which experienced expression of the stemness markers of Sox-2, Oct4, CD117, and CD133 [108]. The above cells exhibited differentially up-regulated expressing NF-kB proteins and selective over-expression of viral oncogenes E6/E7 just in HPV16 +ve cells that established greater numbers of orospheres, over-expressed c-Rel, and selectively actuated p65, which hetero-dimerized with p50 for showing greater DNA binding activities. Moreover, selective overexpressed miR-21 and miR-155 and down-regulation of miR-34a have been revealed via HPV +ve cancer stem cells overexpressing HPV16 oncogene E6, which have control over maintaining stemness. Although HPV-ve CSCs exhibit only p50 homo-dimeriztion, weak differentiations, and the worst prognoses, HPV infections involved participating p65 with de-regulated expression of certain miRNAs resulted in the detailed differentiation of tumors and more acceptable prognosis [108].

### Epigenetic regulators and oral cancer stem cells

#### Histone demethylases

Growing trend of documents show that various cancers such as oral cancers might be modulated in under epigenetic condition via histone demethylases or microRNAs [15, 61, 109–118]. One of the groups of histone demethylases epigenetically regulated transcribing gene via removal of the histone methylation marks [119]. Accordingly, histone demethylases contribute crucially to the dominating gene transcription via modifying chromatin availability and transcriptional machinery. Convincing documents demonstrated that histone demethylases involve in different cellular procedures such as carcinogenesis, cell fate selection, and cell differentiation [120–122]. Currently, the increasing trend of documents showed the essential contribution of histone demethylases such as JARID1, KDM4, LSD1, KDM6B, KDM6A, KMD3, KDM5, and Jumonji domain–consisting of protein 6 (JMJD6) to the cancer stem cell phenotype in several kinds of cancers [61, 123–130].

JMJD6 has been represented as a new molecular modulator of OCSCs [61]. JMJD6 is one of the histone arginine demethylases, which favorably eliminates methyl groups from dimethylated arginine 2 of histone 3 (H3R2me2) and arginine 3 of histone 4 (H4R3me2) [131]. Thus, it leads to the dynamic modulation of transcription. Moreover, JMJD6 modulates expressing gene via modulation of RNA splicing [132], which indicates that JMJD6 is a multi-faceted modulator of the gene expressions. A study showed that JMJD6 improves OSCC cancer stem cell populations; that is, tumor spheres and ALDH1^HIGH^ cell population in comparison with the oral stem cancer cells non-cancer stem cell populations, including adherent mono-layer cells and ALDH1^low^ cell population [61]. It has been shown that silencing JMJD6 caused losing self-renovation potential, migration capability, and chemoresistance in OSCC cells. Moreover, reports indicated that JMJD6 knock-down in aggressive breast cancer cell lines declined cell emigration; however, its over-expression augmented cellular motility [133]. There is an interaction between JMJD6 and the splicing factor U2AF65. JMJD6 regulates alternate splicing of vascular EGF (VEGF) receptor [132]. Moreover, results demonstrated that alternate splicing of the VEGF receptor via U2AF65 enhanced endothelial cell emigration, and JMJD6 silencing in the endothelial cells caused lower emigration [134]. Hence, it is necessary to determine the impacts of JMJD6 on EMT. Contrarily, overexpressing JMJD6 increases both the CSC traits and the numbers of CSCs, which suggests that JMJD6 is a prominent modulator of the cancer stem cell phenotype and genesis in OSCC.

#### MicroRNAs

The increased trend of investigations reflected the utilization of the noncoding RNAs as the upstream regulator of the CSCs using diverse systems like EMT regulation [135, 136].

In fact, the non-coding RNAs represent the RNA, which would not encode a protein and thus may be categorized into multiple groups like the long non-coding RNAs (lncRNAs = above 200 nucleotides in length) and small non-coding RNAs (like microRNAs = approximately 19 to 22 nucleotides in length) [137–141]. Finally, it has been found that that microRNAs (miRNAs) regulated translational effectiveness or stability of the targeted mRNAs via interactions with 3′-un-translated region (3′-UTR) of the respective targets [142–147]. These molecules exert their effects via targeting a variety of molecular and cellular mechanisms [148–152]. Hence, miRNAs could be used as diagnostic, prognostic and therapeutic biomarkers in the treatment of different diseases such as stroke, cancer, cardiovascular diseases, infection diseases, diabetes, and viral infections [153–163].

There is evidence of the significant down-regulation of miR-200c expression in the ALDH1^+^/CD44^+^ HNSCC with greater BMI1 expression level [164]. In addition, researchers demonstrated possible significant inhibition of malignant CSC features or BMI1 knock-down by upregulating the miR-200c could so that ZEB1 or ZEB2 knockdown may enhance the miR-200c and suppress the BMI1 expressions in the ALDH1^+^/CD44^+^ HNSCC cells, which revealed that interactions between ZEB1/ZEB2, BMI1, and miR-200c detected the fate of the cancer stemness in OSCC. Put differently, one of the popular tumor repressors called p53 could attach to the promoter area of the miR-200c at several locations [164]. Moreover, researchers approved that as a most repeatedly in-activated tumor inhibitor gene in HNSCC, losing the level of p53 expression correlated to metastatic ability of HNSCC [165, 166]. Finally, p53 mutation could contribute to down-stream transcriptional actuation of the miR-200c, which enhanced the CSC features.

In addition, some studies showed that ALDH1^+^CD44^+^ HNSCC cells expressed lower level of miR145 and thus inhibiting miR-145 has been adequate for driving the tumor-inducing characteristics in the ALDH1^−^CD44^−^ HNSCC cells [167]. Therefore, the miR-145 could experience a direct binding to the ADAM17 and SOX9 through their 3′-UTR areas. Consequently, analysis showed that SOX9 directly modulated the ADAM17 promoter and this SOX9/ADAM17 axis determined miR-145-mediated CSC and EMT and features. As a result, that mediation of IL-6 and soluble IL-6 receptor secretion by the miR-145-ADAM17 pathway has been revealed that could played a role in maintaining the CSC characteristics in a paracrine way. Curcumin is a natural compounds that could be used in the treatment of different diseases such as cancer [168–175]. The curcumin delivery attenuated the tumor expansion in vivo by enhancing the miR-145 promoter activities [167].

### Calcium channels

Recently, authors proposed the significance of calcium signaling in modulating oral cancer stemness traits [60, 176–178]. Ca^2+^ is one of the global second messengers regulating several physiological procedures, and disruption of its homeostasis would be observed during carcinogenesis, which results in the deregulation of the rapid growth of the cells, emigration, and apoptosis inhibition [179–182]. In a majority of the nonexcitable cells, it has been found that Ca^2+^ influx is strongly modulated via the store-operated Ca^2+^ entry (SOCE) path, and mediated by store-operated Ca^2+^ release-actuated Ca^2+^ (CRAC) channels [183]. Research revealed that Orai1 is one of the crucial pore subunits of CRAC channels [184–186]. When the cells stimulated, they experience releasing Ca^2+^ from the endoplasmic reticulum (ER), accompanied by extra-cellular Ca^2+^ influx via SOCE. However, it should be stated that SOCE both re-fills the depleted ER Ca^2+^ stores and presents a direct Ca^2+^ signal for activating down-stream responses such the nuclear factor of the actuated T-cells (NFAT) signaling path [187, 188]. Researchers have widely examined Orai1 in immunology, because NFAT is a transcription agent with a vital role to activate, differentiate, and effector functions of T-cells [189]. Results indicated essential contribution of Orai1 to carcinogenesis [60, 177, 181, 190–197].

Higher expression of Orai1 is observed in cancer stem cells-improved cell population, including tumor spheres and ALDH1^HIGH^ population of OSCC [60]. Moreover, Orai1 is capable of endowing non-tumorigenic immortalized oral epithelial cells with self-renovation, and concurrently enhances transcribing pluripotent and cancer stem cells-associated agents such as Nanog, Sox2, KLF4, Oct4, Zeb1, Bmi1, and Zeb2. Studies also illustrated that ALDH1^+^ cancer stem cells population in non-tumorigenic oral epithelial cells is increased by expressing ectopic Orai1, which enhances OSCC metastatic potentials. This result agrees with the other publications representing the significance of Orai1 in the emigration capability of invasive breast cancer cells [133]. Suppressing Orai1 in numerous OSCC cell lines resulted in suppressing CSC traits. Therefore, our hypothesis is that Orai1 would promote malignant development of OSCC via enrichment of the CSC phenotype. Yet, there is not enough knowledge of the basic mechanism, through which Orai1 modulates oral cancer stemness.

NFAT is a major down-stream objective of Orai1-mediated Ca^2+^. It is de-phosphorylated via a protein phosphatase complex containing calcineurin and calmodulin [198, 199]. researchers found important contribution of NFAT for maintaining CSCs in human cancers like melanoma pancreatic, colonic, and lung [200–203]. In addition, researchers showed that silencing NFATc3 in the cells having an overexpressed ectopic Orai1 in OSCC cells caused inhibiting CSC phenotype. Moreover, an NFAT chemical suppressor substantially suppressed cancer stemness in cells. Thus, NFATc3 would be necessary for the Orai1-induced CSC phenotype, indicating practical contribution of the Orai1/NFATc3 axis to the oral CSC modulation.

Ca^2+^ oscillation (spatio-temporal modulation calcium signaling) is more crucial compared to the overall modifications in cytosolic Ca^2+^ concentrations in the area of tumor invading nature, progression, and cancer stemness [176–178]. Ca^2+^ oscillation is the final outcome of Orai1-mediated SOCE so that Orai1 would be improved in OCSCs. As suppressing Orai1 channel performance led to the complete shut-down of Ca^2+^ oscillation in OSCC cells, Orai1-mediated Ca^2+^ oscillation might be a potent selective target to treat oral CSCs.

### Other mechanisms

From among the above pathways, researchers largely confirmed contribution Bmi1 and Notch signaling in oral cancer stemness. Activating the Notch1 signaling pathway is necessary to maintain cancer stem cells, and demands attachment of its ligands Jagged 1 (JAG1), JAG 2, and δ-like, accompanied by the proteolytic releases of the Notch intra-cellular domain (NICD), and activating NICD down-stream target genes [204]. We formerly stated that if the OSCC cells are exposed to the pro-inflammatory cytokine TNFα in the long term, they augment self-renovation capacities and tumorigenicity that is related to the actuation of the Notch path [62]. It has been also found that Hes1 in TNFα-induced oral cancer stemness is the objective of the actuated Notch1 so that its knock-down inhibits self-renewing potential of TNFα-treated OSCC cells. Expression of Hes1 is usually performed in multiple un-differentiated cell kinds in the growing mouse embryo. It also contributes critically to maintain progenitor cell fate. Data obtained from the studies showed that Hes1-deficient mice exhibited pre-mature differentiation, subsequent lethality, and progenitor cell depletion [205]. In total, the above results suggested that Notch1–Hes1 axis is one of the newly designed axes to regulate oral CSCs self-renewal.

Bmi1 that is one of members of the polycomb group transcription repressors involves in oral cancer [206, 207]. Recently, researchers revealed the essential contribution of Bmi1 to maintenance of the self-renewal capacities of oral CSCs [208, 209]. In addition, in a case of the use of the genetic lineage tracing, in vivo contribution of Bmi1 to regulate the stemness of oral cancer stem cells such as its self-renewal and tumorigenic potentials has been obviously illustrated [209].

## Oral cancer stem cell therapy

Cancer therapy is very important aspect in the public health field [210–212]. Many researchers developed a variety of therapeutic approaches such as immune cell therapy, stem cell therapy, gene therapy, nanotechnology-based therapy, and utilization of natural compounds in the treatment of various cancers [213–219]. In this regards, several fields emphasize the identification and specific targeting of the neck and neck CSCs (Table 2) [220]. However, the new therapeutic regimes carried considerable morbidities like defacement and functional modifications from surgical operations to the systemic toxicity caused by chemo-therapy as well as radiation-induced consequences due to radio-therapy. In addition, as a result of diverse innate systems, the CSCs frequently resisted to the conventional radiation and chemo-therapy. Such cells have been capable of surviving through treatment and repopulating the tumors with the chemo-radioresistant cells. thus, the specifically targeting head and neck CSCs provided a potent device of the ameliorated cancer outputs by demonstrating the organ conservation and declining the off-target toxicity [220].

**Table 2:** Cancer stem cell targeting in head and neck cancer

Therapeutic target	Compound	Mechanism	Model	References
Nanog	Silencing	Suppresses tumorigenic and CSCs-like abilities	In vitro	[226]
Grp78	Silencing	Inhibits tumor growth and stem cell regulatory proteins i.e., slug and Oct-4	In vitro	[225]
CD44	Silencing	Decreases migration, EMT, and reduces the expression of snail, vimentin, N-cadherin and slug	In vitro	[232]
Inhibiting translation elongation	SVC112	Increases the progression of cell-cycle slows and delay DNA repair following radiation. Improves colony and sphere formation	In vitro	[233]
Let-7d/CDC34 axis	Niclosamide	Induces cell cycle arrest in G1 phase	In vitro, in vivo	[234]
5T4	MEDI0641	Decreases the CSC fraction, and tumor regression	In vivo	[235]
cMET/FZD8	PF-2341066	Decreases tumor initiation, sphere formation, and metastatic spread	In vivo	[236]
CD44v6	Anti-CD44v6 antibody BIWA-IRDye800CW and -Indium-111	Detection of tumor regions and invasive zones	In vivo	[237]
CD44	Radionuclide^186^Re-cmAb (U36)	Dose-limiting myelotoxicity, reduction in tumor size	Human	[238]
ALDH1	Alda-89, Aldi-6	In combination with cisplatin improves apoptosis and decreases tumor growth	In vitro, in vivo	[239]
Porcupine (PORCN) (Wnt signaling)	LGK974	High response in HNSCC with Notch loss of function mutation	In vitro	[240]
FGF	BGJ398	Reduces ALDH^high^CD44^high^, sensitization to cisplatin	In vitro	[241]
Bmi1/AP-1	PTC-209	Cisplatin plus PTC-209 potently eradicates Bmi1 + CSCs and suppresses progression of tumor	In vitro	[209]

As seen in the literature, CD44 is one of the well-known exploration targets for the targeted therapies against CSCs. In fact, researchers utilized hyaluronic acid (with its selective binding to CD44) as one of the agents to deliver the directed treatments as opposed to the CD44 positive cells like the hyaluronic acid conjugated chemo-therapeutics as well as the hyaluronic acid guided NPS. Moreover, hyaluronic acid induced the interactions between CD44 and the stem cell transcription factors Nanog, Sox2, and Oct-4 [58]. Thus, additional investigations should be performed for showing advantages of the hyaluronic acid targeting with any induction of more activation of the CSCs. Consequently, experts in the field explored the anti-CD133 treatments as the targeted head and neck anti-CSC therapies. One of the studies on the bacterial toxin (cyto-lethal distending toxin) to an antihuman CD133 monoclonal antibody revealed inhibiting the cells proliferation while other investigation, which utilized a single-chain variable fragment targeting CD133 demonstrated remarkable diminishment in the rapid growth of the tumors in the cells and rat models [221, 222]. As a result, CD271 inhibited in the cell models for decreasing the formation of the tumors [223]. Finally, one of the encouraging options to treat this condition would be targeting the CSC surface markers and the best performance in relation to the remaining treatments would be as a delivery mechanism.

Notably, one of the main today’s investigation fields is the addition of the novel agents or targeted treatment related to the standard cisplatin chemo-therapy. Moreover, salino-mycin with paclitaxel and cisplatin functioned for increasing apoptosis in the neck and neck CSCs [224]. Additionally, GRP78 has been considered to be one of the multi-functional protein contributed to the cell survival as well as resistance to chemo-therapy. Inhibiting the GRP78 would sensitize the head and neck CSCs for radiation and chemotherapy [225]. In this regard, Huang et al. revealed the greater sensitivity to cisplatin by small hairpin RNA knock-down of Nanog [226]. Furthermore, researchers indicated that CSCs had lower levels of ROS, assisting in the maintenance of the stem-like features and chemo-resistance. Finally, inhibiting the ROS scavenging proteins (SOD2 & Catalase) enhanced the ROS and the following enhancement in the sensitivity to cisplatin [227].

Experts in the field are growingly applying the epidermal growth factor receptor (EGFR) inhibition (with cetuximab) in the advanced and recurring HNSCC therapeutic guidelines. The former investigations also suggested the potent contribution to the EGFR targeted treatment especially as opposed to the head and neck CSCs. However, in the naso-pharyngeal carcinomas, EGFR acted using *CTNNB1* and *AKT* pathways for driving the CSC phenotypes [228]. Moreover, activating EGFR in the head and neck CSCs enhanced expressing the genes engaged in the CSC rapid growth or proliferation (*OCT4, BMI1, CD44, NANOG*) and CSCs treatment through inhibiting EGFR declined the tumor growth and augmented the sensitivity to cisplatin [228].

Greater abilities for the efflux cytotoxic agents have been considered as one of the main devices of CSC resistance to chemo-therapy. Therefore, researchers examined the cellular efflux proteins as the potent targets to sensitize the CSCs to the current chemo-therapy agents. Earlier research on the laryngeal cancer cell-lines diminished the CSC proportion by verapamil that is one of the inhibitors to the ABCG2 membrane transporter [229]. It is notable that suppressors to other members of ABC transporter family in case of application to the head and neck and CSC populations, enhanced sensitivity to the chemo-therapy [230]. Moreover, authors largely explored the enhanced CSC sensitivity to the radiation. However, today’s examinations target ATRA (a retinoid involved in cell terminal differentiation) and *CHEK1/2* DNA damage repair genes in the head and neck CSCs. Such explorations demonstrate greater responses to the radiation in the CSCs following the *CHEK1/2* suppression and ATRA utilization [79]. Finally, inhibiting the *SHH/MTOR/RPS6KB1* pathways augmented radio-sensitivity to CSCs, reflecting the contribution of the above pathways and potent targetable choices to enhance the CSC radio-sensitivity [231].

## Conclusion

One of very important players in the initiation, and progression of cancer are CSCs. A variety of reports documented that these sub-populations of cancer cells are associated to different properties of cancer such as metastasis, tumorigenicity, and recurrence. Hence, CSCs are known as the root of the cancer. Moreover, targeting the CSCs would be one of the encouraging as well as evasive treatment options, which aimed to enhance efficacy and specificity for eradicating the tumors and declining the systemic or off-target toxicity. Consequently, investigations of the additional description and targeted treatments towards the head and neck CSCs would be one of the active and fast growing fields. Given that CSCs exert their tumorigenesis roles via affecting on a sequencing of cellular and molecular targets and pathways (i.e., microRNAs, histone modifications and calcium regulations). Therefore, more and better understanding of CSCs actions can provide unique opportunities to develop new therapeutic platforms for targeting CSCs in the treatment of various cancers.

## Data Availability

The primary data for this study is available from the authors on direct request.

## Abbreviations

ABC: (ATP)-binding cassette
ALDH1: Aldehyde dehydrogenase1
CRAC: Ca^2+^ release-actuated Ca^2^
CSCs: Cancer stem cells
EGF: Epidermal growth factor
EMT: Epithelial-mesenchymal transition
ESA: Epithelial-specific antigen
iPSCs: Induced pluripotent stem cells
JAG1: Jagged 1
HMLEs: Human mammary epithelial cells
HNSCC: Head and neck squamous cell carcinoma
NFAT: Nuclear factor of the actuated T-cells
NICD: Notch intra-cellular domain
OSCC: Oral squamous cell carcinoma
SC: Stem cell
SCID: Severe combined immune-deficient
Zeb: Zinc-finger E-box–binding homeobox
